# Recent progress in postoperative nausea and vomiting after thyroidectomy

**DOI:** 10.3389/fsurg.2026.1897151

**Published:** 2026-07-08

**Authors:** Qihao Zhao, Fuling Song, Meimei Cui, Haoping Jia, Rongzhan Fu, Guanghan Wu, Dongsheng Zhou

**Affiliations:** 1 Department of General Surgery, The First Affiliated Hospital of Shandong First Medical University & Shandong Provincial Qianfoshan Hospital, Jinan, China; 2 School of Clinical Medicine, Shandong Second Medical University, Weifang, China; 3 Department of Cardiology, The First Affiliated Hospital of Shandong First Medical University & Shandong Provincial Qianfoshan Hospital, Jinan, China; 4 School of Traditional Chinese Medicine, Beijing University of Chinese Medicine, Beijing, China; 5 Department of Anesthesiology, the First Affiliated Hospital of Shandong First Medical University & Shandong Provincial Qianfoshan Hospital, Jinan, China

**Keywords:** anesthesia, multimodal prophylaxis, postoperative nausea and vomiting, risk factors, thyroidectomy

## Abstract

Postoperative nausea and vomiting (PONV) is a common and distressing complication following thyroidectomy. Notably, vomiting can increase both the risk and severity of postoperative complications, including cervical hematoma formation, wound dehiscence, postoperative bleeding, increased pain, delayed recovery and, in severe cases, airway compromise. The pathophysiology of PONV is multifactorial, involving interactions among peripheral surgical stimulation, central emetic pathways, anesthetic-related mechanisms and individual patient susceptibility. The recognized risk factors include female sex, non-smoking status, a history of motion sickness or prior PONV and exposure to volatile anesthetics or opioid analgesics during surgery. Contemporary perioperative practice emphasizes evidence-based multimodal prophylactic strategies supported by current consensus guidelines and randomized controlled trials to reduce PONV, including total intravenous anesthesia (TIVA), opioid-sparing analgesic techniques and intraoperative administration of serotonin (5-HT3) receptor antagonists and corticosteroids. Comprehensive risk assessments and individualized antiemetic strategies are essential for effectively reducing the incidence of PONV and improving overall postoperative outcomes in patients undergoing thyroidectomy. This narrative review summarizes recent progress in the epidemiology, mechanisms, risk assessment, prevention and treatment of PONV following thyroidectomy, with particular emphasis on thyroidectomy-specific factors and clinically applicable perioperative strategies. A better understanding of the underlying mechanisms may facilitate individualized risk stratification and optimize patient-centered perioperative care, ultimately improving postoperative outcomes in thyroidectomy patients.

## 1 Introduction

Thyroidectomy is one of the most commonly performed endocrine surgeries worldwide and is inevitably associated with a range of intraoperative and postoperative complications. Different thyroidectomy techniques may be associated with distinct complication profiles (1).

Postoperative nausea and vomiting (PONV) remains a common and distressing adverse effect of anesthesia, typically occurring within the first 24 h after surgery. PONV is classified according to the timing of onset as early (within 6 h postoperatively) or late (6–24 h postoperatively) (2). While the incidence of PONV in the general surgical population is well-documented, thyroidectomy-specific risk factors such as vagal nerve stimulation, neck positioning, and the use of intraoperative neuromonitoring (IONM) have not been sufficiently addressed in the existing literature. PONV may lead to serious complications, including aspiration pneumonia, fluid and electrolyte imbalances and esophageal rupture (2, 3). In the absence of prophylactic treatment, PONV occurs in approximately 30%–40% of patients undergoing general surgery, with the incidence rising to nearly 80% in high-risk individuals (4–7). In patients undergoing thyroidectomy, the incidence of PONV has been reported to be higher than that in the general surgical population, reaching approximately 60%–84% in the absence of effective prophylactic antiemetic strategies or in high-risk cohorts (5, 6, 8). Some studies have estimated that PONV may occur in approximately 70% of adult patients after thyroidectomy. This high incidence may be related to the demographic risk profile of thyroidectomy patients, including the predominance of female and non-smoking patients, as well as thyroidectomy-specific factors such as neck hyperextension, surgical manipulation, and vagal stimulation (9–11). PONV prolongs the average length of stay in post-anesthesia care units and hospitals, increases healthcare costs and reduces patient satisfaction (12–14).

A variety of patient-, anesthesia- and surgery-related factors have been identified as contributors to an increased the risk of PONV, such as a young age, female sex, history of PONV, non-smoking status and a history of severe motion sickness (15). Among surgical populations, patients undergoing thyroidectomy are predominantly non-smoking females, a demographic profile associated with an increased susceptibility to PONV. Although PONV after thyroidectomy generally occurs within the first 24 h and is often transient, it can contribute to postoperative complications, including wound dehiscence, bleeding, neck hematoma and airway obstruction secondary to hematoma formation (16).

This review aims to provide updated guidance for anesthesiologists, endocrine surgeons, and perioperative teams specifically regarding the management of PONV after thyroidectomy. We present an individualized risk stratification approach and propose a targeted prophylactic pathway that incorporates thyroidectomy-specific risk factors, including the role of neck positioning, vagal nerve manipulation, and IONM. By addressing these unique factors, this review fills an important gap in the current literature and provides clinicians with practical strategies to reduce the incidence of PONV and improve patient outcomes in thyroidectomy patients.

## 2 Epidemiological evidence of the causes of PONV following thyroidectomy

### 2.1 Patient-related factors

Studies have shown that the incidence of PONV is mainly influenced by several factors, including sex, age, smoking status and a history of motion sickness (17, 18). The incidence of PONV is higher in females than in males, a difference that may be associated with hormonal influences. However, the incidence of PONV declines in women aged over 50 years, especially among those who are postmenopausal. The incidence of PONV is lower in patients with a history of smoking compared with non-smokers, possibly due to the induction of hepatic cytochrome P450 enzymes by polycyclic aromatic hydrocarbons in cigarette smoke, leading to enhanced metabolism of emetogenic substances (19). The incidence of PONV is higher in patients with a history of motion sickness, which is mainly due to the increased sensitivity of the vestibular function. Upon stimulation, it activates receptors linked to neurotransmitters such as histamine, serotonin and acetylcholine, thereby inducing nausea and vomiting (9).

### 2.2 Anesthesia-related factors

Anesthetic techniques and agents, including opioids, inhalational anesthetics and intravenous general anesthetics, are closely associated with the incidence of PONV. For example, opioids and inhalational anesthetics are among the most common medications causing PONV. The mechanism of opioid-induced PONV is primarily related to the central nervous system effects of opioids. Opioids, such as morphine and fentanyl, are capable of stimulating the chemoreceptor trigger zone (CTZ) and the vomiting center in the floor of the fourth ventricle, thereby contributing to an increased risk of PONV (9). Additionally, opioids may reduce muscle tone, decrease gastrointestinal motility and delay gastric emptying, thereby contributing to an increased risk of PONV (20). Inhalational anesthetics, such as nitrous oxide (N_2_O), halothane, enflurane, isoflurane and sevoflurane, may directly stimulate chemoreceptors, thereby activating the vomiting center and contributing to the development of PONV. The mechanism of N_2_O-associated PONV has not been fully elucidated; potential explanations include increased middle ear pressure, bowel distension, activation of the dopaminergic system in the CTZ and interactions with opioid receptors (21). Inhalational anesthetics, including halothane, enflurane and isoflurane, may modulate autonomic nervous system activity (both sympathetic and parasympathetic), resulting in elevated catecholamine levels and thereby contributing to nausea and vomiting. Furthermore, increasing duration of anesthesia has been associated with a higher incidence of PONV. Apipan et al. indicated that the incidence rates were 9.6%, 22.2%, and 42.6% for anesthesia durations of <2 h, 2–4 h, and >4 h, respectively. These findings suggest that patients receiving inhalational anesthesia for >4 h have an approximately fivefold higher incidence of PONV compared with those with durations of <2 h. These findings indicate a positive association between the duration of inhalational anesthesia and the incidence of PONV (22). Certain intravenous anesthetic agents, including ketamine, etomidate and propofol, may also cause nausea and vomiting.

### 2.3 Body position-related factors

Thyroidectomy often requires the elevation of the shoulders and back, along with excessive neck extension to improve the surgeon's access to the thyroid gland. However, this position may reduce blood flow to the vertebral arteries, which are crucial for the brain's blood supply. The vertebral arteries supply the posterior circulation of the brain, and reduced blood flow during neck hyperextension can activate a range of physiological responses, including dizziness, nausea, and vomiting. A study by Tominaga and Nakahara demonstrated a significant decrease in bilateral neck arterial blood flow velocity during excessive neck extension, which was associated with a higher incidence of postoperative nausea and vomiting (PONV). This suggests that optimizing neck positioning and limiting excessive extension could help reduce PONV by maintaining adequate vertebral artery perfusion during surgery (10). Additionally, traction on the vagus nerve during thyroidectomy may lead to heightened vagal impulses. The release of chemical mediators during surgery activates emetic chemoreceptors, thereby contributing to the development of PONV (11). Moreover, studies have demonstrated a correlation between intraoperative hypotension and the incidence of PONV following thyroidectomy (23).

### 2.4 Surgery-related factors

Recurrent laryngeal nerve palsy (RLNP) is one of the most significant and concerning complications associated with thyroid surgery (24). However, intraoperative neuromonitoring (IONM) has been proposed as a method to ensure and verify the functional integrity of the recurrent laryngeal nerve during thyroid surgery. Some studies have suggested that, following thyroidectomy, patients who undergo IONM may experience a lower quality of recovery, an increased risk of PONV, and higher medical costs (25). However, this could be due to confounding factors, such as prolonged anesthesia duration, rather than a direct physiological effect of IONM itself. Further research is needed to clarify this relationship. Additionally, compared to other surgical procedures, intraoperative traction of the vagus nerve during thyroid surgery may amplify vagal reflex activity, potentially heightening emetogenic triggers.

To improve the clinical applicability of this review, the principal thyroidectomy-specific determinants of postoperative nausea and vomiting are summarized in Table 1, in which patient-, anesthesia-, position-, and surgery-related contributors are linked to their proposed mechanisms and practical implications for perioperative risk stratification. In thyroidectomy, these factors are particularly important because postoperative vomiting may have consequences beyond patient discomfort, including wound dehiscence, bleeding, neck hematoma, and potential airway compromise.

**Table 1:** Thyroidectomy risk factors and mechanisms for postoperative nausea and vomiting.

Category	Risk factor	Mechanism	Relevant evidence
Patient-Related Factors	Gender	Higher incidence in females, potentially related to hormonal levels	Hormonal fluctuations may increase PONV risk (2, 7, 15, 17, 18)
	Age	Higher risk in younger patients	Younger patients have faster metabolism, increasing PONV occurrence (9, 15, 18)
	Smoking History	Lower incidence in smokers	Smoking increases liver cytochrome P450 enzyme activity, helping metabolize emetogenic substances (2, 7, 9, 19)
	History of Motion Sickness	Higher incidence in patients with a history of motion sickness	Vestibular function sensitivity triggers PONV through neurotransmitter receptors such as histamine, serotonin, and acetylcholine (2, 7, 9)
Anesthesia-Related Factors	Anesthetic Type	Inhaled anesthetics (e.g., isoflurane, sevoflurane) and opioids are commonly associated with PONV	Inhaled anesthetics activate the vomiting center, increasing PONV risk (2, 8, 9, 20)
Position-Related Factors	Excessive Neck Extension	Decreases bilateral neck arterial blood flow, triggering nausea and vomiting	Reduction in vertebral artery blood flow leads to PONV (10)
Surgical Manipulation	Vagus Nerve Traction	Traction on the vagus nerve during surgery can increase vomiting reflex	Traction and stimulation of the vagus nerve activates the vomiting center (11, 25)

PONV, postoperative nausea and vomiting.

### 2.5 Application and limitations of established PONV risk scores in thyroidectomy

The Apfel simplified risk score is one of the most commonly used tools for predicting PONV in clinical practice. It includes four independent predictors: female sex, non-smoking status, a history of PONV or motion sickness, and postoperative opioid use (2, 7). The risk of PONV increases as the number of these factors increases. This model is highly relevant to thyroidectomy patients because many patients undergoing thyroid surgery, particularly young or middle-aged female non-smokers, already meet several classic Apfel risk criteria before surgery (15, 17, 18). Therefore, the Apfel score may be useful as a baseline screening tool for identifying patients who require multimodal prophylaxis.

However, the Apfel score was developed for general surgical populations and does not include several thyroidectomy-specific variables. These include neck hyperextension, cervical and vagal manipulation, use of intraoperative neuromonitoring, prolonged anesthesia duration, intraoperative hypotension, and delayed oral intake after surgery (10, 11, 23, 25). These factors may further increase the risk of PONV after thyroidectomy but are not captured by the traditional Apfel score. Therefore, in thyroidectomy patients, the Apfel score should be used as an initial risk assessment tool and supplemented with thyroidectomy-specific risk modifiers. Such an adapted approach may help clinicians provide more individualized and procedure-specific PONV prophylaxis.

## 3 Mechanism of PONV

Nausea and vomiting are essential protective mechanisms that help prevent the ingestion or digestion of potentially harmful substances. It is well-established that the centers regulating nausea and vomiting are located in the medulla oblongata and involve interconnected neuronal networks. During episodes of nausea and vomiting, these neurons are sequentially activated and coordinated into a central pattern generator. Within the medulla oblongata, two distinct functional regions are involved in the vomiting response: the reflex center and the chemoreceptor trigger zone (26).

The mechanism of PONV is triggered by various afferent stimuli, which are ultimately integrated and coordinated by the vomiting center and CTZ (26). Stimulation of receptors located in areas such as the base of the tongue, pharynx, stomach, intestines, gallbladder, bile ducts, and urogenital organs, as well as receptors in the cerebral cortex and vestibular organs, generates excitatory signals that travel via the vagus, sympathetic, and glossopharyngeal nerves to the emetic center in the lateral medulla oblongata (27). In addition, the CTZ contains a variety of receptors, including dopamine, serotonin (5-HT3), histamine, opioid, and cholinergic receptors, which play key roles in the emetic response. These receptors can detect various toxins, metabolites, or drugs in the blood and cerebrospinal fluid, sending signals to the central nervous system. These signals then reach the cerebral cortex, inducing sensations of nausea and dizziness, or propagate through pathways involving the vagus, sympathetic, trigeminal, glossopharyngeal, hypoglossal, and spinal nerves to the digestive tract, diaphragm, and abdominal wall muscles. The transmitted signals trigger coordinated physiological responses, including relaxation of the upper esophageal sphincter, closure of the glottis and posterior nares, as well as intense contractions of the diaphragm and abdominal muscles. These contractions compress the stomach and elevate intragastric pressure. Simultaneously, the sphincters above the stomach and below the esophagus, along with the muscles surrounding the esophagus relax, allowing gastric contents to be expelled through the esophagus and mouth, thereby triggering vomiting (27, 28). In the context of thyroidectomy, PONV should therefore be understood as the result of interactions between peripheral surgical stimuli and central emetic processing. Peripheral triggers may include stimulation of the pharyngeal and laryngeal regions during airway manipulation, traction of cervical tissues and vagal afferent activation during thyroid dissection, whereas central mechanisms may involve activation of the CTZ and vomiting center by volatile anesthetics, opioids and inflammatory mediators.

## 4 Treatment

Research on preventive and therapeutic measures for PONV has been extensive, covering both pharmacological and non-pharmacological interventions. The major preventive and rescue strategies are summarized in Table 2, including their perioperative role, representative timing and dosing patterns, key safety considerations, and the relative strength of the supporting evidence. Although no drugs have been specifically developed for PONV, conventional agents target specific neuro-receptors that play central roles in its pathogenesis. Pharmacological options include dopamine and 5-HT3 receptor antagonists, anticholinergics, antihistamines, glucocorticoids, opioid receptor antagonists, and neurokinin-1 receptor antagonists. Non-pharmacological interventions encompass electroacupuncture, traditional acupuncture, acupressure and adjustments in anesthesia management. However, preventive and therapeutic strategies for post-thyroidectomy nausea and vomiting remain inadequately established. This section will explore several approaches to address this issue.

**Table 2:** Prophylaxis and rescue options for postoperative nausea and vomiting.

Category	Medication/Treatment	Typical Dosing and Timing	Strength of Evidence	Key Safety Concerns
Pharmacological Prevention	5-HT3 Receptor Antagonists (e.g., Ondansetron)	4–8 mg, 30 min before surgery	Strong evidence (2, 3, 8, 30, 31)	May prolong QT interval, caution in patients with underlying cardiac disease
	Corticosteroids (e.g., Dexamethasone)	4–8 mg, 30 min before surgery	Strong evidence (2, 34–36)	Risk of hyperglycemia, osteoporosis, and use caution in patients with diabetes or hypertension
	Opioid Antagonists (e.g., Naloxone)	0.4–2 mg, postoperative use	Moderate evidence (2, 3, 20)	Overuse may reduce analgesic effects
Non-Pharmacological Treatment	Electroacupuncture	Applied within 24 h post-surgery	Moderate evidence (58, 59)	No significant side effects
	High-Concentration Oxygen	Adjust oxygen concentration as needed	Preliminary evidence (60)	Potential impact on swallowing and parathyroid function
	IONM	Used to protect the vagus nerve during surgery	Strong evidence (25, 61)	Increases recovery costs but improves recovery quality
	OFA	Multimodal regimen including *α*-2 agonists, N-methyl-D-aspartate receptor antagonists, local anesthetics	Strong evidence (49, 51, 52)	May require careful monitoring, potential for increased costs
	TIVA	Propofol, remifentanil	Strong evidence (5, 8, 51)	Risk of hypotension, respiratory depression

5-HT3, 5-hydroxytryptamine type 3; IONM, intraoperative neuromonitoring; OFA, opioid-free anesthesia; TIVA, total intravenous anesthesia.

### 4.1 Pharmacological prevention and treatment

#### 4.1.1 5-HT3 receptor antagonists

5-HT3 receptor antagonists selectively block presynaptic 5-HT3 receptors in the vagal afferents of the gastrointestinal tract and CTZ, thereby inhibiting the neural transmission of emetic signals to the vomiting center and suppressing nausea and vomiting (28, 29). Commonly used potent 5-HT3 receptor antagonists include ondansetron, ramosetron, azasetron, tropisetron and palonosetron. These antagonists are characterized by their safety, low cost, and efficacy, making them the first-line medications for the prevention and treatment of PONV (30). However, these agents may prolong the QT interval and should be used with caution in susceptible patients, especially those with underlying cardiac conditions or other risk factors for arrhythmia (31). Through a network meta-analysis, Cho *et al*. demonstrated that propofol and tropisetron, both individually and in combination, as well as the combination of ramosetron and dexamethasone, effectively prevent PONV, PON, and POV in patients undergoing thyroidectomy. These combinations reduce the need for rescue antiemetics and improve the complete response rate (8). Additionally, Lee *et al*. demonstrated that the combination of ramosetron and dexamethasone significantly reduces the incidence of nausea, the need for rescue antiemetics, 1-h postoperative pain scores on the verbal rating scale (VRS), ketorolac use, and the occurrence of shivering in thyroidectomy patients, compared to ramosetron alone (32). A prospective randomized controlled study by Joe et al. indicated that the combination of total intravenous anesthesia (TIVA) with prophylactic ramosetron significantly reduces the incidence of early PONV and severe late postoperative vomiting, compared to sevoflurane anesthesia (33).

#### 4.1.2 Glucocorticoids

The antiemetic effects of commonly used glucocorticoids, such as dexamethasone and methylprednisolone, are thought to be mediated by the inhibition of inflammatory mediator release and the stabilization of cellular membranes. Tarantino et al. confirmed that a single-dose regimen of dexamethasone offers effective and safe prophylaxis against PONV in patients undergoing surgery for papillary thyroid carcinoma (34, 35). Notably, Ye et al. also found that a single preoperative dose of 8–10 mg of dexamethasone effectively reduces both the incidence of PONV and postoperative pain in thyroidectomy patients (36). However, due to the potential side effects of glucocorticoids, caution should be exercised when administering them to patients with conditions such as pregnancy, osteoporosis, diabetes, or severe hypertension. For other patients undergoing thyroidectomy, the administration of dexamethasone prior to anesthesia induction is recommended, as it is primarily suited for the prevention, rather than the treatment, of PONV (37).

#### 4.1.3 Anticholinergic drugs

The mechanism of anticholinergic drugs involves inhibiting the binding of acetylcholine to cholinergic receptors in smooth muscle, peripheral ganglia, and the central nervous system, thereby disrupting signal transmission. Commonly used agents include atropine and scopolamine. A recent study indicated that combining penehyclidine with TIVA using propofol-remifentanil more effectively prevents PONV than TIVA alone, particularly within 2–24 h after total thyroidectomy (38). Notably, despite potential adverse reactions such as dry mouth, drowsiness, dizziness, and impaired cognitive function, recent evidence indicates no significant occurrences of dizziness, drowsiness, or headache, nor any prolongation of the post-anesthesia care unit stay or extubation time, further supporting its favorable safety profile (39).

#### 4.1.4 Other drugs

A study by Choi et al. found that adjuvant use of intraoperative dexmedetomidine infusion may be effective in prevention PONV (40). Propofol-based TIVA has been shown to significantly improve recovery quality in female patients following thyroidectomy compared with desflurane anesthesia (41). Additionally, intravenous lidocaine has been shown to reduce postoperative pain and nausea after thyroidectomy, while enhancing the overall recovery quality (42).

Non-pharmacological interventions, while low-risk and potentially providing short-term relief, show mixed evidence for their effectiveness. Studies on electroacupuncture suggest promising results, but many trials have small sample sizes and limitations. Acupressure on the P6 point can reduce nausea but is not consistently effective for vomiting. High-concentration oxygen may help alleviate PONV, but its impact remains under investigation. Further research is needed to more thoroughly evaluate the effectiveness of these interventions in preventing PONV.

### 4.2 Non-pharmacological prevention and treatment

#### 4.2.1 Carbohydrate drinks

Some researchers consider perioperative fluid balance a key risk factor for PONV, although the impact of preoperative carbohydrate intake remains uncertain. In a prospective randomized study involving 52 non-diabetic thyroidectomy patients, Rajan et al. found that those who did not receive a carbohydrate drink 2 h before surgery had significantly higher intraoperative blood glucose levels and a greater incidence and severity of PONV. However, the intensive care unit stay was comparable to that of patients who received the carbohydrate drink (43).

#### 4.2.2 Anesthesia method

Opioid-free anesthesia (OFA) utilizes a multimodal regimen of *α*-2 agonists, *N*-methyl-D-aspartate receptor antagonists, and local anesthetics to provide analgesia without the use of opioids (44, 45). Current research suggests that OFA can effectively reduce PONV (46–48), particularly in patients undergoing thyroidectomy (49–52). Additionally, TIVA has been demonstrated to reduce the incidence of PONV in patients undergoing total thyroidectomy (53, 54). The aforementioned studies also demonstrate that propofol, as an intravenous anesthetic, can reduce the incidence of PONV. Regional anesthetic techniques have been shown to decrease postoperative pain and reduce the requirement for opioid use following thyroidectomy (55, 56). A recent study indicated that bilateral superficial cervical plexus block provides enhanced postoperative analgesia, reduces opioid consumption, decreases PONV, and improves visual analogue scale (VAS) pain scores (57).

#### 4.2.3 Acupoint therapy

Acupoint therapy is one of the most commonly used non-pharmacological interventions, with the Nei-Guan (P6) acupoint being the most extensively studied. Research has shown that P6 acupressure provides short-term relief from nausea, although it does not significantly reduce vomiting or retching (58). Simultaneously, electroacupuncture treatment after thyroidectomy has been shown to be safe and comparable to conventional antiemetic therapy (59).

#### 4.2.4 Other methods

Due to the need for shoulder and back elevation and neck hyperextension during thyroidectomy, preoperative thyroid position training is implemented to help acclimatie patients to the neck hyperextension position, potentially reducing the incidence of PONV. However, evidence supporting its efficacy is limited, and further research is needed to confirm its effectiveness. Additionally, efforts should be made to avoid intraoperative hypotension in patients. Zhou et al. reported that high-concentration oxygen may reduce PONV and postoperative pain following thyroidectomy and may be associated with less severe voice changes. However, its impact on swallowing and parathyroid function requires further investigation (60). Postoperative high-concentration oxygen may be considered a potential adjunct after thyroid surgery; however, thyroidectomy-specific evidence remains limited, and its effects on swallowing and parathyroid function require further study. Moreover, a study has suggested that combining IONM with evidence-based nursing markedly improves postoperative recovery and the quality of life in patients with thyroid cancer (61).

## 5 Conclusions

PONV is a common adverse reaction following surgery that significantly affects patients' quality of life and recovery. The occurrence of PONV reflects the interplay of multiple mechanisms influenced by surgical manipulation, anesthetic techniques, and patient-related factors. Prevention strategies include preoperative assessment, appropriate anesthetic selection, and intraoperative fluid management. For patients experiencing PONV, treatment methods such as oral or intravenous antiemetics can be employed. A deeper mechanistic understanding of PONV is essential for improving postoperative outcomes. Future research should focus on the following areas. Firstly, the development of standardized outcome definitions for PONV, including clearer criteria for early vs. late PONV, severity scales, and rescue antiemetic needs. Secondly, the comparative effectiveness of bundled prophylaxis strategies in thyroidectomy cohorts, particularly those integrating pharmacological agents with non-pharmacological therapies like acupuncture or high-concentration oxygen. Lastly, mechanistic studies examining the effects of vagal nerve stimulation and neck positioning on PONV, which remain poorly understood in the current literature. Addressing these research gaps will help refine PONV management strategies and improve the overall quality of recovery in thyroidectomy patients.
